# Gephyrin: a scaffold that builds a phase at the inhibitory postsynapses

**DOI:** 10.1038/s41422-020-00440-2

**Published:** 2020-11-17

**Authors:** Christian Hoffmann, Dragomir Milovanovic

**Affiliations:** Laboratory of Molecular Neuroscience, German Center for Neurodegenerative Diseases (DZNE), 10117 Berlin, Germany

**Keywords:** Membrane trafficking, Molecular biology

**The scaffolding protein gephyrin is a core component of many inhibitory synapses. Bai and colleagues now show that phase separation of gephyrin with the subunits of both glycine and GABA receptors underlies the formation of postsynaptic sheets at the inhibitory synapses**.

To assure precise processing of information, contacts between neurons require the alignment of the release sites, presynaptic terminals, with the ionotropic transmitter receptors at the postsynaptic plasma membrane. Downstream of these receptors, scaffold molecules, kinases and cytoskeleton components all orchestrate the signaling cascade induced by the neurotransmitter binding and ion influx. Each neuron in the mammalian central nervous system relies on both the excitatory and inhibitory inputs. A long-standing view is that the postsynaptic density (PSD) represents a hallmark of the excitatory synapses. Recent cryo-electron tomography work suggested that even the inhibitory synapses contain thin-sheet densities of ~5 nm, so-called iPSD, underneath the clusters of glycine receptor (GlyR) and type-A γ-aminobutyric acid receptor (GABA_A_R).^1^

It is emerging that the principles of liquid-liquid phase separation (LLPS) could explain the mechanisms behind the formation and regulation of the PSD at the excitatory synapses.^2,3^ LLPS is a process in which one or multiple components in the same state segregate from another component into distinct compartments, for example, demixing of oil in water. In the context of cell biology, it is a process where (bio)polymers separate from homogenous aqueous mixtures forming fluid condensates.^4^ In the study published in *Cell Research*, Bai and colleagues examine whether the principles of LLPS might underlie the formation of iPSDs,^5^ focusing on the interaction between transmembrane receptors and gephyrin, a well-known scaffold protein of inhibitory synapses.

**Fig. 1:**
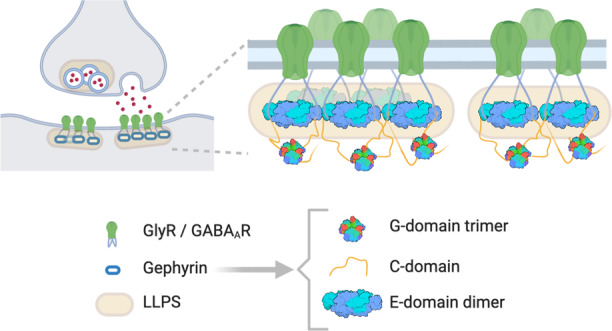
Scheme of gephyrin-mediated liquid condensation at the inhibitory postsynapse. LLPS (yellow) is induced by the interaction of gephyrin (blue) with the subunits of glycine and GABA_A_ receptors (GlyR and GABA_A_R, respectively; green). Gephyrin consists of G-domain (PDBID: 1IHC) and E-domain (PDBID: 2FU3) linked by a flexible C-domain. Scheme was generated with BioRender.

Gephyrin is essential for the clustering of both GlyR and GABA_A_R^6^ and contains an N-terminal trimerization G-domain (GPHN-G) and a C-terminal dimerization E-domain (GPHN-E) linked by a flexible C-domain. GlyR and GABA_A_R, via their intracellular loops, bind to the E-domain of gephyrin.^7^ Traditionally, gephyrin is considered to form hexagonal lattice crosslinking the receptors at the plasma membrane and connecting them with the cytoskeleton and downstream signaling molecules.^7^ Here, the authors show that the loop of GlyR β subunit fused to GCN4 coiled-coil (GlyR-β) and E-domain of gephyrin (GPHN-E) form liquid condensates in solution (at low µM concentrations) and at the supported membrane (in the nM range).^5^ The ability of GlyR-β to oligomerize plays an important role in its capability to phase separate with GPHN-E. Besides GlyR-β, TM3–4 loops of GABA_A_R α3-subunit also phase separates with GPHN-E suggesting liquid condensation to be a general feature of inhibitory synapses (Fig. 1). The phase separation occurs at the equimolar ratio of receptors and scaffold proteins, a range similar to their physiological concentrations.^8^

There are several implications of this study. First, the observation that different regions of the same proteins (GlyR-β and GPHN-E) are important for phase separation or direct binding, suggests that these two processes could be regulated separately. The positively charged region of GlyR-β (a.a. 354–383) and negatively charged surface at the subdomain II of GPHN-E are essential for condensate formation, despite hydrophobic interactions being critical for their direct binding. Indeed, the authors show that increasing salt concentration does not disrupt the binding between GlyR-β and GPHN-E, but it prevents phase separation. Moreover, the full-length gephyrin has a reduced ability to form liquid condensates. The authors mapped two inhibitory peptide sequences (a.a. 259–274 and a.a. 303–318) within the linker region of the C-domain. Interestingly, the dynein light chain, which is known to interact precisely with the C-domain of gephyrin,^9^ is sequestered in the liquid phase of gephyrin and GlyR-β. This implies that the same proteins can act both as scaffolds (to form a core of liquid condensate) and as a bait to recruit partner proteins for specific downstream biological function. Finally, this study breaks with the dogma that PSDs are only a feature of excitatory synapses. The authors reconstitute the proteinaceous sheets observed at the inhibitory synapses and show them to be formed by a similar mechanism as disc-shaped structures at the excitatory postsynapse.

As it often happens, this study opens a range of interesting questions. What is the effect of phosphorylation on the gephyrin-mediated phase? The authors generated phosphomutants in different regions of gephyrin’s C-domain, showing that phosphorylation of various sites can either enhance or reduce the condensate formation. Further studies will be needed to identify how specific kinases regulate these different phosphorylation sites during neuronal activity. Another critical point is that GABA_A_R subunits can cluster independently of gephyrin,^10^ implying that other factors and scaffold proteins, e.g., collybistin,^11^ might be the additional mediators of the postsynaptic phase formation at the inhibitory synapses. Finally, in pathology, some patients with autism spectrum disorder, schizophrenia and epilepsy contain mutations in *GPHN* gene that encodes the G-domain of gephyrin.^12^ It remains to be determined to what extent these mutations and deletions of gephyrin alter its phase separation properties and potentially modulate the molecular basis of these complex diseases.
